# Research on the decoupling effect and driving factors of industrial carbon emissions in Hubei province

**DOI:** 10.1038/s41598-025-93277-x

**Published:** 2025-03-21

**Authors:** Hongxing Tu, Songtao Xu, Peiwen Tu, Xu Xiao

**Affiliations:** 1 Economics and Management School, Hubei Polytechnic University, Huangshi, 435003 Hubei China; 2 Center for Cognitive Science and Transdisciplinary Studies, Jiujiang University, Jiujiang, 332005 Jiangxi China; 3 Bangor College, Central South University of Forestry & Technology, Changsha, 410004 Hunan China; 4 Business School, Central South University, Changsha, 410083 Hunan China

**Keywords:** Carbon emission, Decoupling analysis, LMDI, Hubei industry, Energy and society, Environmental economics, Environmental impact, Psychology and behaviour, Socioeconomic scenarios, Sustainability

## Abstract

Under the “dual carbon” goal, the new quality productivity in the industrial energy sector will become an important force in promoting the green and high-quality development of Hubei Province’s economy and society. The article comprehensively uses the Tapio decoupling model and LMDI decomposition method, and empirically analyzes the decoupling effect and driving factors of industrial carbon emissions in Hubei Province from 2006 to 2022 using panel data of industrial industries. Research has found that the growth of Hubei’s industrial economy and carbon emissions have undergone a fluctuating process of "strong decoupling → weak decoupling → expansion negative decoupling". For a considerable period of time in the future, the industrial economic growth and carbon emissions in Hubei will still be in a weak or expanding negative decoupling state. From the decomposition results, it can be seen that the trend of changes in the energy intensity index and carbon intensity index shows a high degree of consistency, and the energy intensity index has become the main factor driving the decrease in Hubei’s industrial carbon intensity index. However, the impact of energy structure effects and industrial structure effects on industrial carbon emissions is relatively weak, and the dividends brought by structural effects are still not significant. On this basis, the article proposes relevant policy recommendations, providing theoretical basis and policy basis for empowering high-quality industrial development in Hubei Province with new quality productivity in the post epidemic era.

## Introduction

Currently, in the context of global efforts to address climate change, studying the complex relationship between economic development and carbon emissions has always been a focus of attention for academia and decision-makers. As a pillar industry of the national economy, industry contributes greatly to energy consumption and carbon emissions. The success of industrial emission reduction is directly related to the smooth achievement of China’s "carbon peak and carbon neutrality" goals. As one of the world’s largest carbon emitters, China’s carbon accounting database shows that in 2022, China’s cumulative carbon emissions reached 11 billion tons, accounting for approximately 28.87% of the global carbon emissions. Among them, industrial emissions amounted to 4.2 billion tons, accounting for 38.18% of the national emissions. According to statistics from the Ministry of Industry and Information Technology of China, the energy consumption in the industrial sector accounts for about 65% of the total national consumption. Among them, high energy consuming manufacturing industries such as chemical, steel, and cement account for 35% of China’s total carbon emissions, making the industrial sector an important area for energy conservation and carbon reduction. Currently, it has been less than 10 years since China’s goal of peaking carbon emissions by 2030. However, many studies have shown that China has not yet achieved decoupling between industrial economic growth and carbon dioxide emissions, and the energy driven economic growth model still persists in industries such as electricity, steel, cement, and coal chemica^1–6^. How to effectively reduce industrial carbon emissions, especially in the high-energy consumption and high emission manufacturing industry, will be the key to achieving China’s “dual carbon” goals on time.

Hubei Province is one of the important old industrial bases in China and an important pivot for implementing the “Central Rise” strategy proposed by the Chinese government, with an extremely important strategic position. In 2022, the equipment manufacturing industry in Hubei Province achieved a revenue of 1.9652 trillion yuan, accounting for 30.4% of the total industry in Hubei Province and ranking 7th domestically. However, from the perspective of carbon source structure, the total amount of carbon sources in Hubei Province in 2018 was 141.44 million tons. Among them, the energy consumption of carbon sources is 138.19 million tons, accounting for 97.7% of the total carbon sources, and industrial energy consumption remains the largest carbon source. But it is gratifying that Hubei Province formulated and issued the "Interim Measures for the Management and Trading of Carbon Emission Rights" as early as 2014. In terms of carbon market construction, the carbon trading volume and trading amount have always been among the top in pilot provinces and cities in China. According to the information released by the Department of Ecology and Environment of Hubei Province, from 2016 to 2022, the cumulative carbon emissions per unit of GDP in Hubei Province have decreased by 23.9%, and both the carbon emissions per unit of GDP and per capita carbon emissions are lower than the national average. As of June 30, 2023, the cumulative transaction volume of Hubei’s carbon market quota secondary market is 365 million tons, with a transaction volume of 8.831 billion yuan, maintaining a leading level in the pilot carbon market. In summary, Hubei Province has made positive progress in controlling total carbon emissions, carbon sinks, and carbon market trading. However, there is still huge room for improvement in optimizing the carbon source structure, and the situation of energy conservation and emission reduction in industry is not optimistic.

As a new, efficient, and sustainable form of productivity, new quality productivity has been included for the first time in the 2024 Chinese government work report. The new quality productivity has the characteristics of high technology, high efficiency, and high quality, and conforms to the advanced productivity quality of the new development concept. The important statement that 'new quality productivity itself is green productivity’ elucidates that the development of new quality productivity can provide a new driving force for the realization of China’s' dual carbon 'goals. Therefore, developing new quality productive forces in the industrial energy sector will become an important force in promoting high-quality economic and social development in Hubei Province, and is also an important path to achieving Hubei’s “dual carbon” goals. Based on the above background, this article comprehensively applies the Tapio model and LMDI decomposition technology to explore the decoupling effect of industrial carbon emissions in Hubei and its driving factors, providing theoretical support and policy guidance for promoting high-quality industrial development in Hubei and building a “Beautiful Hubei” in the post epidemic era. The contribution of this study to existing literature research is mainly reflected in: (1) Compared with existing research, this paper not only measures the decoupling effect between industrial economic growth and carbon emissions in Hubei, but also attempts to analyze the main influencing factors of carbon intensity changes, enriching the existing literature research methods; (2) There are not many comprehensive studies on industrial carbon emissions in Hubei Province. The research results of this article will provide theoretical and policy basis for empowering high-quality development of Hubei’s industry with new quality productivity in the post epidemic era, the perspective of the topic selection is innovative.

The remainder of this paper is structured as follows: Sect “Literature review” presents a literature review. Sect “Methods and data” introduces the model setting and variables selection for the study. Sect “Empirical results” describes the findings of empirical testing and analyzes the results. Sect “Conclusion and discussion” summarizes our conclusions and associated policy implications. And finally, Sect “Further summary and discussion” presents the research limitations and outlook.

## Literature review

Based on the analysis of existing literature, we found that there are roughly two types of research on carbon emissions: one is to use the Tapio model to measure the decoupling relationship between economic growth and carbon emissions. Decoupling analysis can reflect the real-time dynamic relationship between economic growth and environmental pressure, and can quantitatively measure the dependence of economic growth on carbon emissions. This analysis method has been widely used in the study of economic growth and environmental issues^7^. The decoupling theory was first proposed by the Organization for Economic Cooperation and Development to describe the fundamental theory of blocking the link between economic growth and resource consumption (or environmental pollution). At the end of the twentieth century, the OECD introduced the concept of decoupling into agricultural policy research and gradually expanded it to areas such as the environment. Subsequently, scholars from different countries have widely applied decoupling theory to various fields such as transportation, tourism, industry, electricity, and agriculture, and have achieved fruitful research results^8–12^. For example, when studying the relationship between European economic development and carbon emissions between 1970 and 2001, Tapio used decoupling elasticity to construct decoupling indicators, effectively overcoming the problem of the OECD decoupling index model’s difficulty in determining the base period^13^. Freitas’ research found that Brazil’s economic activity and carbon dioxide emissions changed in the opposite direction between 2004 and 2009, with a clear decoupling trend between the two^14^. Hatzigeorgiu used a decoupling analysis model to study the causal relationship between Greece’s gross domestic product and carbon dioxide emissions^15^.

Another type is to use various decomposition techniques based on decoupling analysis to decompose the factors that affect carbon emissions, in order to facilitate precise policy implementation by policy makers^16^. The decoupling analysis model was introduced to China relatively late, but in recent years, using this method to analyze the issue of high-quality economic development in China has formed fruitful research results^17,18^. Among them, the most representative one is Chen, who decomposed the main reasons for the changes in carbon dioxide intensity in China’s entire industrial sector since the reform and opening up, and found that the decrease in energy intensity or the increase in energy productivity are the determining factors for the fluctuation and decrease in carbon dioxide emission intensity^19^. Tu and Xiao used data from industrial enterprises above a certain scale in 30 provinces and cities in China to analyze the sources of China’s industrial economic growth using environmental production frontier functions^20^. Wang and Hu introduced the intermediate variable of energy-saving elasticity to construct the Tapio decoupling model, and conducted empirical research on the CO_2_ decoupling elasticity and energy-saving elasticity of 28 manufacturing industries in China^21^. Tu et al. used the Tapio model to analyze the decoupling relationship between China’s industrial economic growth and carbon emissions from 1994 to 2010, as well as the influencing factors of carbon intensity changes^22^. Fang et al. used the spatial Durbin model to study the spatiotemporal evolution patterns of green innovation and urban carbon emission efficiency, the results showed that green innovation has a positive and significant impact on the carbon emission efficiency of Chinese cities^23^. Zha et al. introduced the logarithmic mean Dirichlet index method and the dynamic effects analysis method based on vector autoregressive model into the decoupling index model, and empirically analyzed the decoupling relationship between tourism growth and carbon emissions in Chengdu, China^24^. Wang and Li et al. studied the decoupling relationship between power generation and carbon dioxide emissions in various provinces of China, and analyzed the driving factors of decoupling^11^. Wang and Hu et al. proposed a carbon emission optimization strategy for the national land space of Hubei Province based on the calculation of total carbon sources and sinks using land use in Hubei Province^25^. Yi and Niu established a VRA model based on data from Hubei Province from 1986 to 2019 to analyze the impact and impact of development mode and industrial structure on carbon emissions in Hubei Province^26^. Wang and Guo et al. comprehensively used the Tapio decoupling index and LMDI decomposition method to calculate the decoupling index of economic development and carbon dioxide emissions from 2000 to 2019 at the overall and inter provincial levels in China, and analyzed the driving factors of decoupling^27^.

Through reviewing the above literature, we found that existing studies have explored the complex relationship between economic growth and carbon dioxide emissions from different perspectives, but most of the studies are still limited to the economic, national or sectoral level, especially in Hubei Province, which has been hit most by the COVID-19 epidemic. Based on the above background, this article uses statistical data from 33 large-scale industrial industries in Hubei Province from 2006 to 2022 to comprehensively analyze the decoupling status and driving factors of industrial carbon emissions in Hubei Province, providing theoretical support and policy guidance for promoting high-quality development of Hubei’s industry in the post epidemic era, and enriching existing research on new quality productivity from a practical perspective.

## Methods and data

### Tapio decoupling model

Decoupling is a commonly used concept in physics, which originally referred to the early release of the response relationship between two or more physical quantities. Later, it was introduced into the field of environmental economics. Carter (1966) first used this physical concept to analyze the response relationship between resource consumption and economic development^28^. Tapio (2005) introduced the concept of elasticity in economics into decoupling theory and further proposed the concept of decoupling elasticity^13^. Due to Tapio’s use of decoupling elasticity to construct decoupling indicators, it can effectively overcome the dilemma of the OECD decoupling index model in base period selection^9^. Therefore, based on Tapio’s research ideas, this article attempts to decompose the causal relationship between industrial economic growth and carbon emissions in Hubei, and introduces energy consumption (E) as an intermediate variable to construct a decoupling theoretical model between industrial carbon emissions and economic growth in Hubei. Decompose the decoupling elasticity index between industrial carbon emissions (CO_2_) and total industrial output(G) into an emission decoupling factor between carbon emissions and energy consumption ε(CO_2_, E), as well as the energy consumption decoupling factor between energy consumption and total industrial output value ε(E,G), the specific decomposition process is as follows:

1 $$\begin{gathered} \varepsilon \left( {CO_{2} ,G} \right) = \frac{{\% \Delta CO_{2} }}{\% \Delta G} = \frac{{\% \Delta CO_{2} }}{\% \Delta E} \times \frac{\% \Delta E}{{\% \Delta G}} \hfill \\ \varepsilon \left( {CO_{2} ,E} \right) = \frac{{\% \Delta CO_{2} }}{\% \Delta E} = {{\frac{{\Delta CO_{2} }}{{CO_{2} }}} \mathord{\left/ {\vphantom {{\frac{{\Delta CO_{2} }}{{CO_{2} }}} {\frac{\Delta E}{E}}}} \right. \kern-0pt} {\frac{\Delta E}{E}}} \hfill \\ \varepsilon \left( {E,G} \right) = \frac{\% \Delta E}{{\% \Delta G}} = {{\frac{\Delta E}{E}} \mathord{\left/ {\vphantom {{\frac{\Delta E}{E}} {\frac{\Delta G}{G}}}} \right. \kern-0pt} {\frac{\Delta G}{G}}} \hfill \\ \end{gathered}$$

**Fig. 1:**
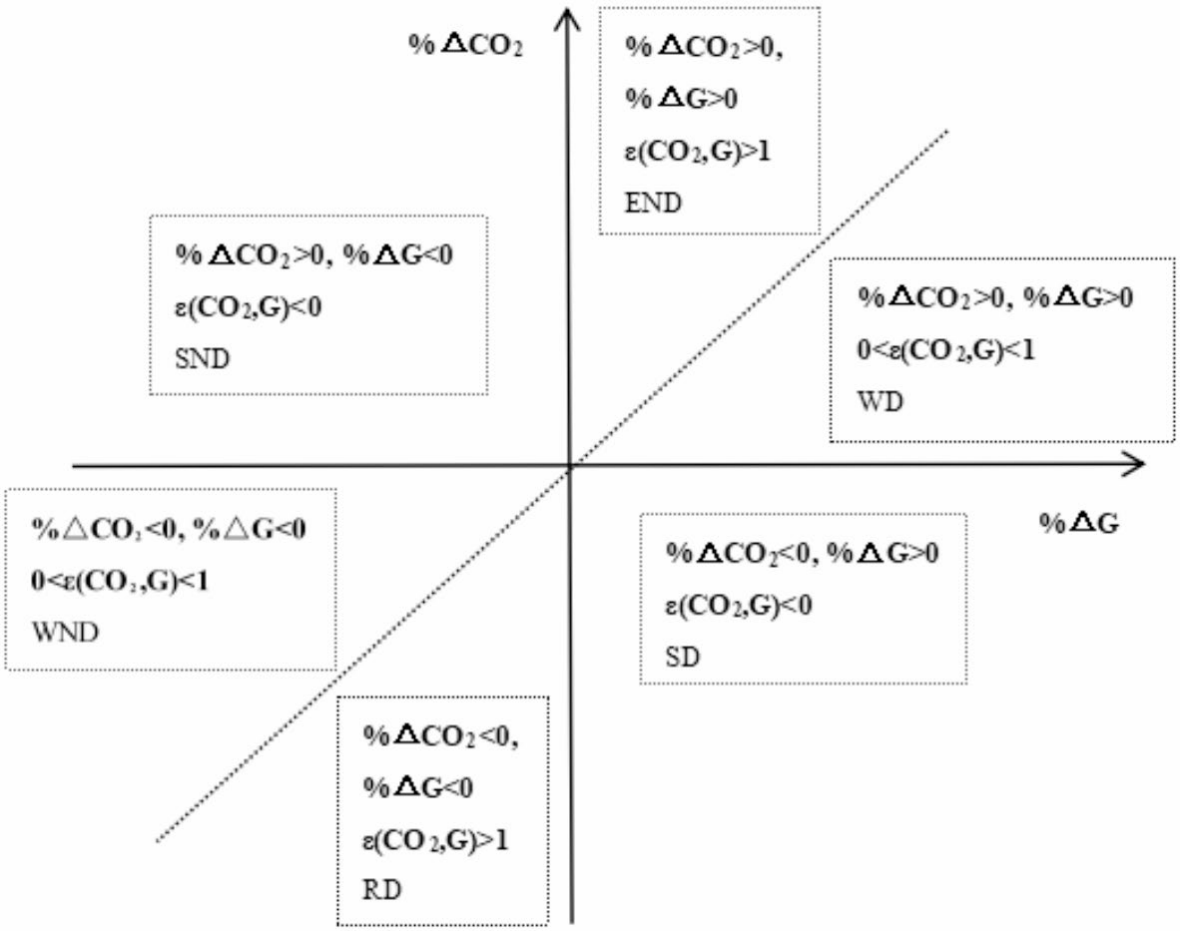
Classification and evaluation criteria for decoupling state. *END* expansion negative decoupling, *WD* weak decoupling, *SD* strong decoupling, *RD* recessive decoupling, *WND* weak negative decoupling, *SND* strong negative decoupling.

Drawing on Tapio’s research approach, this article divides the decoupling of economic growth and carbon emissions into three states: negative decoupling, decoupling, and connectivity. Based on the magnitude of elasticity, environmental pressure, and changes in economic growth, it is further subdivided into six decoupling states^13^. The classification and evaluation criteria for these states are constructed as shown in Fig. 1.

### Logarithmic mean divisia index (LMDI)

In order to further explain the reasons for the decoupling of carbon emissions in the industrial industry in Hubei Province, this article uses the LMDI product decomposition method to factor decompose the intensity of carbon dioxide emissions. Let Y, C, and CI represent industrial added value, carbon dioxide emissions, and their intensity, *i* and *j* represent 33 double-digit industries and 3 types of primary energy (i.e.,raw coal, crude oil, and natural gas). C_ij_, E_ij_, EC_ij_, and ES_ij_ represent the carbon dioxide emissions, energy consumption, carbon dioxide emission coefficients, and energy consumption type structure of the *j*-th type of energy in the *i*-th industry, respectively. E_i_, Y_i_, EI_i_, and S_i_ represent the energy consumption, industrial output value, energy intensity,and industrial structure of the *i*-th industry. Based on Kaya’s identity, we construct a formula for the carbon dioxide emission intensity of the entire industry:

2 $$\begin{gathered} CI = \frac{{\sum {\sum\nolimits_{ij} {C_{ij} } } }}{Y} = \sum {\sum\nolimits_{ij} {\frac{{C_{ij} }}{{E_{ij} }}} } \cdot \frac{{E_{ij} }}{{E_{i} }} \cdot \frac{{E_{i} }}{{Y_{i} }} \cdot \frac{{Y_{i} }}{Y} \hfill \\ \quad \; = \sum {\sum\nolimits_{ij} {EC_{ij} \cdot ES_{ij} \cdot EI_{i} \cdot S_{i} } } \hfill \\ \end{gathered}$$

Define the symmetric logarithmic weight equation as follows:

3 $$L\left( {a,b} \right) = \left\{ {\begin{array}{*{20}c} {{{\left( {a - b} \right)} \mathord{\left/ {\vphantom {{\left( {a - b} \right)} {\left( {\ln a - \ln b} \right)}}} \right. \kern-0pt} {\left( {\ln a - \ln b} \right)}}\quad a \ne b} \\ {\quad \quad \quad \;a\;\;\quad \quad \quad \quad {\kern 1pt} a = b} \\ \end{array} } \right.$$

So, according to the LMDI product decomposition method, the carbon dioxide emission intensity index of the entire industry can be decomposed into the following four influencing factor terms:

4 $$\begin{gathered} RCI = \frac{{CI_{t} }}{{CI_{t - 1} }} = RCI_{ec} \cdot RCI_{es} \cdot RCI_{ei} \cdot RCI_{s} \hfill \\ = \exp \left[ {\sum {\sum\nolimits_{ij} {\frac{{{{CI_{ij}^{t} } \mathord{\left/ {\vphantom {{CI_{ij}^{t} } {Y^{t} }}} \right. \kern-0pt} {Y^{t} }} - {{CI_{ij}^{t - 1} } \mathord{\left/ {\vphantom {{CI_{ij}^{t - 1} } {Y^{t - 1} }}} \right. \kern-0pt} {Y^{t - 1} }}}}{{Ln\left( {{{CI_{ij}^{t} } \mathord{\left/ {\vphantom {{CI_{ij}^{t} } {Y^{t} }}} \right. \kern-0pt} {Y^{t} }}} \right) - Ln\left( {{{CI_{ij}^{t - 1} } \mathord{\left/ {\vphantom {{CI_{ij}^{t - 1} } {Y^{t - 1} }}} \right. \kern-0pt} {Y^{t - 1} }}} \right)}}Ln\left( {\frac{{EC_{ij}^{t} }}{{EC_{ij}^{t - 1} }}} \right)} } } \right] \cdot \hfill \\ {\kern 1pt} \;\;\;\exp \left[ {\sum {\sum\nolimits_{ij} {\frac{{{{CI_{ij}^{t} } \mathord{\left/ {\vphantom {{CI_{ij}^{t} } {Y^{t} }}} \right. \kern-0pt} {Y^{t} }} - {{CI_{ij}^{t - 1} } \mathord{\left/ {\vphantom {{CI_{ij}^{t - 1} } {Y^{t - 1} }}} \right. \kern-0pt} {Y^{t - 1} }}}}{{Ln\left( {{{CI_{ij}^{t} } \mathord{\left/ {\vphantom {{CI_{ij}^{t} } {Y^{t} }}} \right. \kern-0pt} {Y^{t} }}} \right) - Ln\left( {{{CI_{ij}^{t - 1} } \mathord{\left/ {\vphantom {{CI_{ij}^{t - 1} } {Y^{t - 1} }}} \right. \kern-0pt} {Y^{t - 1} }}} \right)}}Ln\left( {\frac{{ES_{ij}^{t} }}{{ES_{ij}^{t - 1} }}} \right)} } } \right] \cdot \hfill \\ {\kern 1pt} \;\;\;\exp \left[ {\sum {\sum\nolimits_{ij} {\frac{{{{CI_{ij}^{t} } \mathord{\left/ {\vphantom {{CI_{ij}^{t} } {Y^{t} }}} \right. \kern-0pt} {Y^{t} }} - {{CI_{ij}^{t - 1} } \mathord{\left/ {\vphantom {{CI_{ij}^{t - 1} } {Y^{t - 1} }}} \right. \kern-0pt} {Y^{t - 1} }}}}{{Ln\left( {{{CI_{ij}^{t} } \mathord{\left/ {\vphantom {{CI_{ij}^{t} } {Y^{t} }}} \right. \kern-0pt} {Y^{t} }}} \right) - Ln\left( {{{CI_{ij}^{t - 1} } \mathord{\left/ {\vphantom {{CI_{ij}^{t - 1} } {Y^{t - 1} }}} \right. \kern-0pt} {Y^{t - 1} }}} \right)}}Ln\left( {\frac{{EI_{i}^{t} }}{{EI_{i}^{t - 1} }}} \right)} } } \right] \cdot \hfill \\ \;{\kern 1pt} \;\;\exp \left[ {\sum {\sum\nolimits_{ij} {\frac{{{{CI_{ij}^{t} } \mathord{\left/ {\vphantom {{CI_{ij}^{t} } {Y^{t} }}} \right. \kern-0pt} {Y^{t} }} - {{CI_{ij}^{t - 1} } \mathord{\left/ {\vphantom {{CI_{ij}^{t - 1} } {Y^{t - 1} }}} \right. \kern-0pt} {Y^{t - 1} }}}}{{Ln\left( {{{CI_{ij}^{t} } \mathord{\left/ {\vphantom {{CI_{ij}^{t} } {Y^{t} }}} \right. \kern-0pt} {Y^{t} }}} \right) - Ln\left( {{{CI_{ij}^{t - 1} } \mathord{\left/ {\vphantom {{CI_{ij}^{t - 1} } {Y^{t - 1} }}} \right. \kern-0pt} {Y^{t - 1} }}} \right)}}Ln\left( {\frac{{S_{i}^{t} }}{{S_{i}^{t - 1} }}} \right)} } } \right] \hfill \\ \end{gathered}$$

In the above equation, t and t-1 represent adjacent periods, RCI represents the total development index of carbon intensity, and RCI_ec_, RCI_es_, RCI_ei_, and RCI_s_ are decomposed into four factor month on month development indices, namely the carbon emission coefficient index, energy structure index, energy intensity index, and industrial structure index. It is not difficult to find that some studies decompose multiple factors, which not only leads to difficulties in compatibility interpretation, but also the decomposition results still do not exceed the factors of strength effect, structure effect, and scale effect^19^. Based on this, this article decomposes the change in carbon intensity into two main factors: intensity effect and structural effect, with the latter further divided into two factors: energy structure and industrial structure. Due to the fact that the carbon emission coefficients of the three primary energy sources used to calculate carbon dioxide emissions in this article are fixed and unchanged, the carbon emission coefficient index RCI_ec_ on the right side of the equation is equal to 1. In fact, only the last three terms are ultimately decomposed.

### Data source

The main variables involved in this article are the 33 double-digit industrial output value, energy consumption, and carbon dioxide emissions in Hubei Province. The industrial output value and energy consumption data are sourced from the statistical data related to Hubei industry in the Hubei Statistical Yearbook^30^, China Statistical Yearbook^31^, and China Energy Statistical Yearbook over the years. Various statistical yearbooks do not directly provide data on carbon dioxide emissions and require estimation. Due to the inconsistency of energy consumption units, it is necessary to convert them to the unified heat unit standard coal for China’s energy measurement. The conversion coefficients of various energy conversion standard coal are provided by the China Energy Statistical Yearbook. Given that the measurement technology for carbon emissions at home and abroad is already mature^29^, the estimated carbon dioxide emission coefficients for various energy sources used in this article are shown in Table 1.

**Table 1:** Estimation of carbon dioxide emission coefficient for various energy sources.

Energy (unit)	NCV (kilojoules per kilogram or per cubic meter)	CEF (kilogram per gigajoule)	COF	Reference coefficient for converting various energy sources into standard coal in China (kilogram standard coal per kilogram or per cubic meter)	Estimation coefficient of carbon dioxide emissions in China (Kilogram per kilogram of standard coal)
Raw coal	20,908	26	0.99	0.7143	2.763
Crude oil	41,816	20	1	1.4286	2.145
Natural gas	38,931	15.3	1	1.33	1.642

## Empirical results

### Calculation of industrial carbon dioxide emissions in Hubei Province

According to a World Bank report, over 70% of carbon dioxide emissions come from fossil fuel combustion. Therefore, based on the three main primary fossil fuel energy sources (coal, crude oil, and natural gas), and according to the reference method provided by the Intergovernmental Panel on Climate Change(IPCC) of the United Nations, the total carbon dioxide emissions can be estimated by adding up the amount of carbon dioxide emissions caused by various energy consumption. The specific calculation formula is as follows:

5 $$C_{t} = \sum\limits_{i = 1}^{3} {C_{i,t} } = \sum\limits_{i = 1}^{3} {E_{i,t} } \times NCV_{i} \times CEF_{i} \times COF_{i} \times \left( {{{44} \mathord{\left/ {\vphantom {{44} {12}}} \right. \kern-0pt} {12}}} \right)$$

In the above equation, C represents the estimated carbon dioxide emissions, *i* = 1,2,3 represents the three primary energy sources, and E represents their consumption. NCV is the net calorific value, CEF is the carbon emission factor, COF is the carbon oxidation factor, and 44 and 12 are the molecular weights of carbon dioxide and carbon, respectively.

**Fig. 2:**
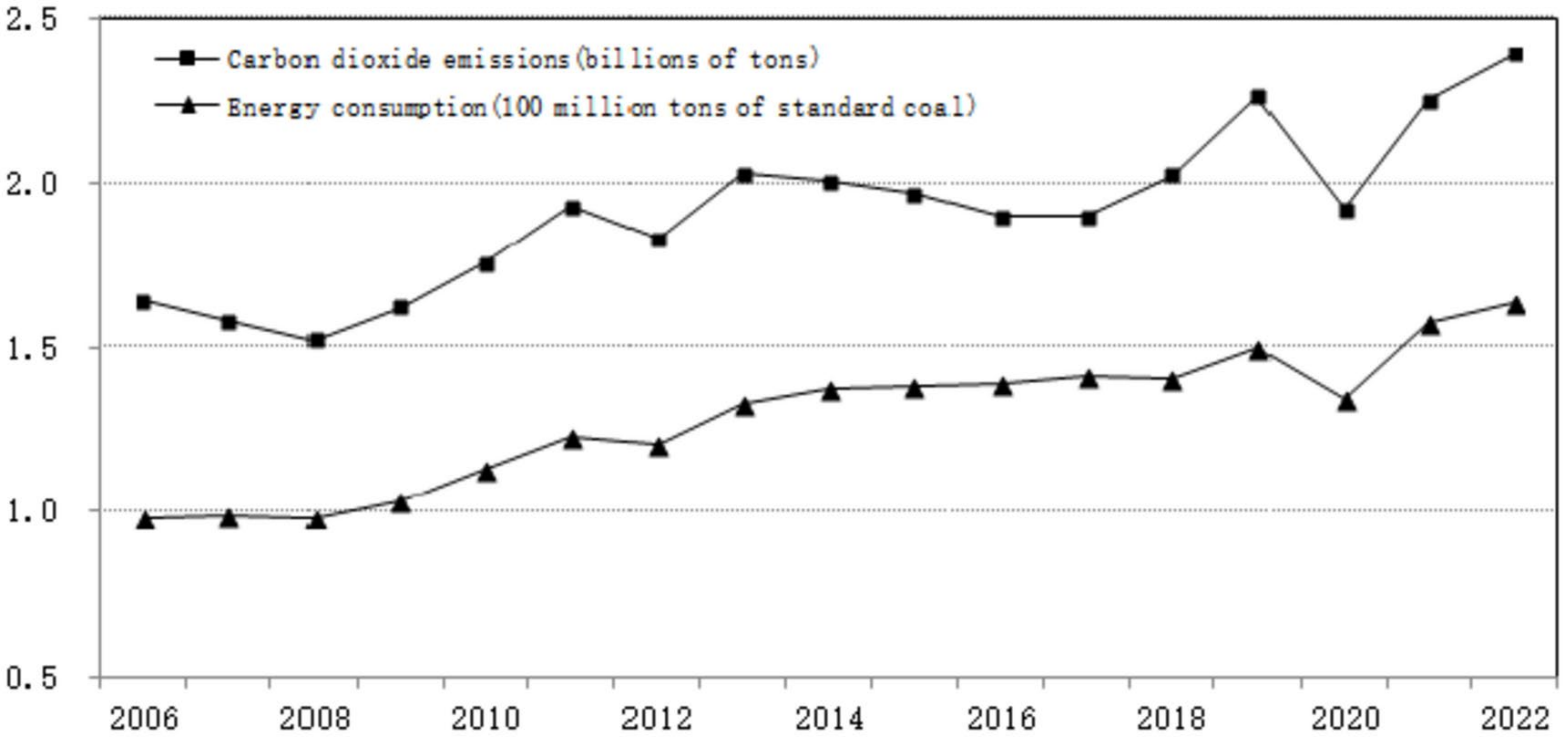
Estimation and comparison of industrial energy consumption and carbon dioxide emissions in Hubei province (2006–2022).

Figure 2 shows the calculation results of energy consumption and carbon dioxide emissions in the industrial sector of Hubei Province from 2005 to 2021. From the perspective of time evolution trends, the energy consumption and carbon emissions of the industrial industry in Hubei have roughly gone through three stages: steady growth, slow decline, and fluctuating increase, and their changing trends are basically consistent. Among them, industrial carbon emissions in Hubei Province have skyrocketed from 157 million tons in 2005 to 226 million tons in 2021, an increase of 43.9%. Before 2011, industrial energy and carbon emissions in Hubei Province had been in a fluctuating upward trend, and there was a brief decline in 2012. Until 2020, the COVID-19, which broke out in Wuhan, will have a huge impact on the entire industry of Hubei, and industrial energy consumption and carbon emissions will decline precipitously. In the first half of 2021, with the end of the COVID-19 and the rapid resumption of production of industrial enterprises in Hubei, industrial energy consumption and carbon emissions show a short-term retaliatory growth trend, and all the way up.

### Analysis of decoupling status of industrial carbon emissions in Hubei Province

From the decoupling status data in Table 2, it can be seen that from 2006 to 2022, the decoupling status of Hubei’s industrial economic growth and carbon emissions has roughly gone through a fluctuating process from strong decoupling to weak decoupling, then to strong decoupling, and finally to recessionary decoupling. From 2006 to 2012, Hubei’s industrial economic growth and carbon emissions remained weakly decoupled for a long time, with both industrial output value and carbon emissions increasing at the same time, but the former grew at a slower rate than the latter. Until 2012, this situation changed. Especially in 2016, despite a negative growth rate of 3.69% in industrial carbon emissions in Hubei, the total industrial output value still maintained a positive growth rate of 6.06%, showing a relatively ideal strong decoupling state. After 2017, the overall growth rate of Hubei’s industrial economy slowed down, especially due to the impact of the COVID-19 in 2020, the total industrial output value of Hubei showed a negative growth of 11.24%, and the entire industrial production of Hubei suffered a serious setback. As the COVID-19 gradually subsided, Hubei’s industrial capacity was rapidly released, and the gross industrial output value and carbon emissions showed a retaliatory growth. In 2021, the regional gross domestic product of Hubei Province exceeded 5 trillion yuan, and Hubei’s industry supported a growth rate of 19.94% in total industrial output value at the cost of 14.71% in carbon emissions, leading to a transition from expansion negative decoupling to weak decoupling between Hubei’s industrial economic growth and carbon emissions. However, just one year later in 2022, facing macroeconomic downward pressure such as insufficient investment growth momentum, weak consumption growth, and external environmental uncertainty, the growth rate of Hubei’s industrial economy has significantly slowed down, but the growth rate of industrial carbon emissions is even faster. Hubei’s industrial economic growth and carbon emissions have returned to a recessionary decoupling state. It can be seen that the decoupling of industrial economic growth and carbon emissions in Hubei has roughly gone through a fluctuating process of "strong decoupling → weak decoupling → expansion negative decoupling", presenting various complex decoupling and re-coupling states. Especially in the long time after the COVID-19 in 2020, Hubei’s industrial economic growth and carbon emissions will be in a weak or expanding negative decoupling state for a long time.

**Table 2:** Analysis of decoupling status of industrial carbon emissions in Hubei province (2006–2022).

Year	%△CO_2_	%△E	%△G	ε(CO_2_,E)	ε(E,G)	ε(CO_2_,G)	Decoupling state
2006	0.0458	0.0719	0.1861	0.6377	0.3863	0.2463	WD
2007	− 0.0392	0.0040	0.2237	− 9.8552	0.0178	− 0.1751	SD
2008	− 0.0387	− 0.0019	0.2864	20.7342	− 0.0065	− 0.1353	SD
2009	0.0617	0.0466	0.1357	1.3232	0.3437	0.4547	WD
2010	0.0769	0.0881	0.2801	0.8722	0.3146	0.2744	WD
2011	0.0905	0.0827	0.2297	1.0943	0.3601	0.3940	WD
2012	− 0.0545	− 0.0203	0.1608	2.6877	− 0.1260	− 0.3387	SD
2013	0.0973	0.0921	0.1469	1.0564	0.6274	0.6628	WD
2014	− 0.0113	0.0331	0.0964	− 0.3423	0.3434	− 0.1175	SD
2015	− 0.0183	0.0044	0.0527	− 4.1252	0.0842	− 0.3474	SD
2016	− 0.0369	0.0091	0.0606	− 4.0622	0.1499	− 0.6090	SD
2017	0.0004	0.0145	− 0.0684	0.0249	− 0.2115	− 0.0053	SND
2018	0.0619	− 0.0060	0.0027	− 10.2478	− 2.2531	23.0892	END
2019	0.1038	0.0629	0.0380	1.6491	1.6569	2.7324	END
2020	− 0.1773	− 0.1152	− 0.1124	1.5395	1.0252	1.5783	RD
2021	0.1471	0.1443	0.1994	1.0198	0.7236	0.7379	WD
2022	0.0579	0.0359	0.0059	1.6107	6.0897	9.8089	END

### Analysis on the causes of carbon emission decoupling in Hubei Industries

**Fig. 3:**
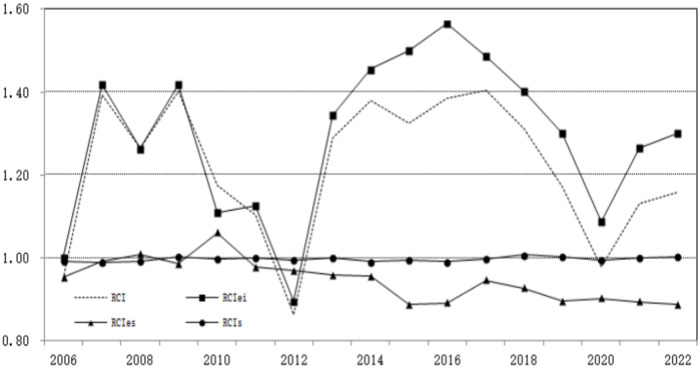
Development index of industrial carbon dioxide emission intensity of Hubei industries and its factor effect decomposition (2006–2022).

Figure 3 depicts the trend of the month on month development index and decomposition index of carbon dioxide emissions intensity in Hubei’s industrial sector. As shown in the diagram, the industrial carbon emissions in Hubei have roughly gone through three stages: slow growth, sharp decline, and fluctuating rise. Among them, the carbon intensity index fluctuates greatly, and its change pattern shows a high similarity with the energy intensity index, with most of the two index curves overlapping over time. Only after 2013, when there were significant changes in the energy and industrial structure, did the difference between the two index curves become much greater. During the research period of 2010, the energy structure index fluctuated and rose to its highest level in history. However, due to the rapid decline of the energy intensity index, there was a brief fluctuation and decline in the industrial carbon intensity index in Hubei from 2009 to 2012.

From the decomposition results in Table 3, it can be seen that the changes in Hubei’s industrial carbon emission intensity index are more explained by the energy intensity index, followed by the energy structure index. The correlation between the industrial structure index and the carbon intensity index is weak. The consumption of fossil fuels is the main factor affecting industrial carbon emissions in Hubei. The reduction of carbon emission intensity fundamentally depends on the reduction of energy intensity or the improvement of energy productivity, which is closely related to the policy orientation of energy conservation and emission reduction in Hubei’s industry. Compared with the energy intensity effect, the impact of energy structure effect and industrial structure effect on carbon emission intensity is relatively weak. Currently, China remains the world’s largest consumer of coal, accounting for 74.2% of primary energy consumption. In 2021, the total energy consumption in Hubei Province was 157 million tons of standard coal, accounting for 2.99% of the total energy consumption in China. Among them, coal consumption accounts for 53.5% of primary energy consumption. It can be seen that as a traditional coal consuming province, Hubei’s industrial development still belongs to the energy driven economic growth model, and it is difficult to reverse this pattern in the short term. Therefore, there is not much room for maneuver in achieving emission reduction targets by adjusting the energy structure. During the 14th Five Year Plan period, if Hubei Province can achieve the expected goal of non fossil energy accounting for more than 20% of total energy consumption, the energy structure effect will highlight the driving effect of emission reduction.

**Table 3:** Cross period average of Hubei industrial carbon intensity index and its LMDI factor decomposition.

Period interval	RCI	Decomposition results		
		RCI_ei_	RCI_es_	RCI_s_
2006–2009	1.2504	1.2757	0.9853	0.9948
2010–2012	1.0447	1.0440	1.0027	0.9980
2013–2016	1.3474	1.4665	0.9237	0.9947
2017–2020	1.2145	1.3199	0.9188	1.0016
2021–2022	1.1453	1.2833	0.8911	1.0017
2006–2022	1.2200	1.2910	0.9471	0.9977

From the perspective of temporal evolution trend, changes in energy intensity are the most critical factor driving the reduction of industrial carbon intensity in Hubei. The direct emission reduction effect brought about by the overall decline in energy intensity is beginning to emerge, and this positive force is gradually strengthening. This indicates that in the past decade, Hubei Province has greatly improved energy production efficiency and directly promoted carbon reduction in Hubei’s industry by encouraging environmental technology innovation and upgrading traditional manufacturing industries. As a traditional manufacturing province, energy consumption remains the engine driving industrial economic growth, and this dependence is also increasing. Therefore, encouraging the development of green technologies to reduce energy intensity and improve energy utilization efficiency is a practical choice for achieving sustainable industrial development in Hubei province.

## Conclusion and discussion

This article comprehensively uses the Tapio model and LMDI decomposition technology to systematically analyze the decoupling relationship between industrial economic growth and carbon emissions in Hubei Province, as well as its inherent driving factors. The research results indicate that the decoupling state between industrial economic growth and carbon emissions in Hubei has roughly gone through a fluctuating process of "strong decoupling → weak decoupling → expansion negative decoupling". For a considerable period of time in the future, the industrial economic growth and carbon emissions in Hubei will still be in a weak or expanding negative decoupling state. The rapid development of the industrial economy comes at the cost of sustained growth in carbon emissions, and achieving the goal of strong decoupling is a long and arduous task. From the decomposition results, energy intensity is the most important factor affecting industrial carbon emissions in Hubei, while the impact of energy structure effects and industrial structure effects on industrial carbon emissions is relatively weak. The policy implications of this article are as follows:

1. Hubei’s industry has not yet achieved coordinated development between economy and environment, and industrial economic growth still relies on the carrying capacity of resources and environment. Although the development of Hubei’s industrial economy has undergone a brief process of strong decoupling. However, with the resurgence of China’s heavy chemical industrialization trend in the twenty-first century, the rapid growth of traditional energy intensive industries has led to a sustained increase in industrial carbon emissions. For a considerable period of time in the future, Hubei’s industrial economy will maintain a strong growth momentum, but this will also contribute to the growth rate of industrial carbon emissions. Hubei’s industrial economic growth and carbon emissions will remain in a weak or expansion negative decoupling state for a long time.
2. The energy intensity index is the most important factor driving changes in industrial carbon intensity in Hubei. Improving energy production efficiency and reducing energy intensity in the short term are practical and feasible paths to promote industrial carbon reduction in Hubei. Relevant statistical data shows that the current energy production efficiency in Hubei Province is less than 40%, and there is great room for improvement in the future. This requires the government departments of Hubei Province to accelerate the establishment of a new energy price formation mechanism, establish a sound carbon trading and carbon finance market system, policy environment, and supporting services, and improve the awareness and participation of enterprises and institutions in the carbon market. Reflecting resource scarcity and environmental governance costs through energy prices, relying on price leverage and market mechanisms to fundamentally promote the improvement of energy production efficiency.
3. The overall impact of energy structure effects on the carbon intensity of Hubei’s industry is relatively small, but in the long run, optimizing the energy structure remains an accelerator for the low-carbon transformation and development of Hubei’s industry. As a major hydropower province, Hubei Province has a natural endowment of water resources in adjusting its energy structure. In 2023, the installed power generation capacity in Hubei Province exceeded 100 million kilowatts for the first time, with clean energy accounting for over 64%. It can be seen that Hubei Province has a good advantage in resource endowment and has inherent conditions for developing clean energy, new energy, and renewable energy. Moreover, Hubei Province has abundant scientific and educational resources, and has established a new energy technology innovation system through the combination of industry, academia, and research, relying on technological innovation to drive the improvement of energy production efficiency.
4. Similarly, the impact of industrial structure effects on the reduction of industrial carbon intensity in Hubei is not significant. Upgrading industrial structure has always been an effective way to reduce energy intensity. Hubei Province has a superior geographical location and has always been known as the "thoroughfare of nine provinces" since ancient times. It has the ability to develop a modern service industry based on smart logistics and multimodal transport. By vigorously developing modern service industries, especially productive service industries, it gradually optimizes the industrial structure and guides the flow of production factors from energy intensive and emission intensive industries to technology intensive light industry and high-tech industries. This is the only way for Hubei Province to transform its industrial development mode. Of course, whether it is energy structure adjustment or industrial structure adjustment, they are not problems that can be solved overnight. It is more appropriate to adopt a gradual approach, and a one size fits all approach will only harm the sustainable development of Hubei’s industry.

### Further summary and discussion

Due to the commission of relevant government decision-making departments in Hubei Province, this article selects Hubei Province as the main research object and carries out a meaningful research work. The research results will provide theoretical basis and policy basis for empowering high-quality industrial development in Hubei Province with new quality productivity in the post epidemic era. However, due to the influence of many uncontrollable factors, there are still some limitations in this study, which need to be further explored in future research. For example, the COVID-19 outbreak in Wuhan, Hubei Province in 2020 may have an impact on relevant statistical data, thus affecting our research conclusions. New quality productivity is characterized by a significant increase in total factor productivity. However, the research conducted in this article is limited to the practical application of new quality productivity in the field of industrial energy, and it cannot cover the rich connotations and extensions of new quality productivity. Further exploration is needed from the perspectives of production, factors, and technology to promote high-quality industrial development. How to find the optimal energy-saving and emission reduction path between maintaining stable industrial economic growth and reducing emissions in response to the current industrial development status in Hubei will also be an important direction for the theoretical research of new quality productivity in the future industrial field.

## Data Availability

Data will be available from the corresponding author upon reasonable request.
